# A Vote of Confidence

**Published:** 1891-04-04

**Authors:** 


### The Hospital

A WEEKLY JOURNAL OF

**Science, Medicine, Nursing, and Philanthropy.**

*For Hospitals, Asylums, Infirmaries, Dispensaries, Medical Practitioners, Students, Nurses, and the Charitable Public.*

VOL. X.—No. 236.] [APRIL 4, 1891.

### A VOTE OF CONFIDENCE.

THE Royal College of Physicians has decided by ninety-six votes, as against seven distributed amongst five other Fellows of the College, to re-elect Sir Andrew Clark President for the ensuing year. This certainly does not mean that there are not other Fellows who are eminently fitted for the dignified position of President. It is well known that there are not a few who would, and in due course will confer honour upon this high medical office. It does, however, mean that Sir Andrew Clark has acted in his capacity as President of the Royal College of Physicians as he has acted in every other capacity throughout his long and honourable life. Sir Andrew may have, and no doubt has, peculiarities, and even faults; and it is a very good thing he has. If he had none, all the rest of us who follow the profession of medicine would cut very sorry figures by comparison with him. Happily for us, Sir Andrew is human, and can therefore err.

It is of much more importance than some people think that the President of the College of Physicians, and indeed the recognised head of any of the great professions, should be something more than a mere figure-head. The present is a time of pulling up, and pulling down. Of pulling up all sorts of men and institutions and asking what may be their right to exist, and to play the part they do; and of pulling down ruthlessly and at once all those men and institutions whose titles are defective or fraudulent. This is not only entirely right, but it is imperative. When hundreds of brave men are pressing to the front of life's battles, it would be madness to give the leadership of our armies to wax models. The College of Physicians ought to have for its head a man of keen vision into the nature of his surroundings, of true sympathy with the intellectual movements of the times, of judgment to discriminate not only between the essentially true and the essentially false, but also between the essentially temporary and the essentially permanent. It should have, too, a man of patient tact and persuasive reasonableness. It is easy to imagine that a mad bull may be quite justified in rushing at a gate and shivering it to atoms by the energy of his impetus. But it would be better both for the bull and the gate if the animal had the sense to lift the latch and walk through. And it is quite possible to conceive that there may be fools among the members and fellows of the College of Physicians; fools which an honest but blundering President might wither with his sarcasm or annihilate with his logic. But it is infinitely to be preferred that the sword of sarcasm shall be kept within its sheath at the College, and that the logic shall be of the clear and persuasive kind which turns dullness into luminosity and enemies into friends.

Sir Andrew Clark is a past master of the art of persuasive logic. Some people attribute this to him as a quality of questionable virtue. They are certainly wrong. It is a quality which is essential for his present delicate position, and it is clearly a quality which the fellows of the College know well how to value. The practically unanimous vote which was given at the Comitia the other evening makes it obvious that Sir Andrew's value is understood. It must not be supposed, however, that tact and reasoning power are his only or even his chief qualifications. We have reason to believe that from the date of Sir Andrew's first appointment, three years ago, until the present time, he has never missed being present at a single meeting of the College, and he has never been even so much as a minute late. These may seem comparatively small performances to take special note of. But who that has had any experience of public affairs has not learnt to value the man who is always surely at his post on the appointed day? And who does not know the still greater value of him who not only keeps his engagement to the day, but is always taking his seat at the committee table as the hand of the clock points to the chosen hour? Attention to business makes a man of business: so long ago as Solomon's time was it said, "He that is diligent in his business shall stand before kings."

It would be a great encouragement and satisfaction to all the thoughtful members of the medical profession if it could be felt that the London College of Physicians was its wise and unprejudiced head, and its sympathetic heart. The Fellows may take it from us that many of the worthiest members of the profession feel themselves placed in a position of unhappy isolation. "Tempora mutantur: nos et mutamur in illis." It must be recognised, not only that the times are changing, but that we are changing with them. A High Court of Appeal is wanted in the medical profession: a court of professional brotherhood, which can inspire every honest-hearted doctor with the assurance that in the last resort he would receive at its hands sympathy and justice.

What are the courts of appeal to which medical men are now compelled to turn? Do they turn to the General Medical Council? They instinctively avoid it! They turn to the professional newspapers. Far be it from us to undervalue any of the literary organs of the medical profession: we could say much for them: we will say nothing against them,

But no single editorial mind, or combination of editorial minds, should have the unchecked power of blasting a reputation or raising an unworthy name to honour. Reputations have been blasted; honourable men have gone down to dishonoured graves; time-servers and flatterers have been raised to wealth and fame; and all by the favouring breath or the chilling contempt of professional editors. This should not be possible. We have often pointed out in these pages that the work of the average medical man is of a domestic kind; much of it lies in the home. Medical men thus often acquire, quite unconsciously to themselves, a timid and half feminine spirit. Is it not the case that they frequently bicker and quarrel among themselves in an inconsequent kind of way, about trifles, after the fashion of women rather than after the fashion of men? This is perhaps inevitable; but if it be inevitable, is not that the very reason why such men should have a trusted Court of Appeal, which thoroughly understands all the inner secrets of professional life, and can judge discriminatingly and justly among them?

There is a feeling among many medical men that the College of Physicians is anxious to incur the guilt of schism. Its Fellows and Members labour to put a gulf between themselves and the great body of practitioners. Of course this is stupidity, absurdity, and every other foolish thing to which a name can be given. From the point of view of the interests of the Fellows and members themselves, it is sheer midsummer madness of the most inconsequent and inexplicable kind. An army may do something without its general, for there are other generals to be found; but what can the general do without his army? The College of Physicians should be the head of the medical profession, not the cocoa-nut at which every ill-conditioned fellow is anxious to have a "shy." It has done well, in that it has chosen Sir Andrew Clark once more for its President; but it will do better still if it takes advantage of his able leadership to identify itself with the interests of the whole profession; to take measures for improving the status of all classes of practitioners, for ameliorating their sterner pressures, and for making the lot of the average doctor more happy, and therefore more successful. Governments and authorities cannot often do very much for those over whom they exercise authority and rule; but they can always do something, and the something which the College of Physicians can do for the profession, it should do forthwith. It never was, and is never likely to be, in a better position for generally popular work, than it is at the present time.

There is in all men, no doubt, a feeling that until they are separated from the great mass of their fellows by some distinguishing mark they have failed to prove the strength of their own character, and almost even to justify their existence. There are many ways by which a man may distinguish himself. The highest and the noblest is the rendering of conspicuous services to others. The higher the position of a man or a public body, the greater is the obligation imposed to act for the public and general advantage rather than for personal interests. The necessities of the average medical man, and the increasingly manifest elevation of what may be called the "tone of the times," plead urgently with the College of Physicians to abandon its attitude of half-friendly isolation, and to place itself frankly at the head of the great body of practitioners as their defender and intelligently sympathetic guide.

